# Prevalence and associated factors of overweight and obesity among persons with type 2 diabetes in Africa: a systematic review and meta-analysis

**DOI:** 10.1080/07853890.2023.2182909

**Published:** 2023-02-23

**Authors:** Emmanuel Ekpor, Samuel Akyirem, Precious Adade Duodu

**Affiliations:** a School of Nursing, University of Ghana, Legon, Ghana; b St. Martins de Porres Hospital, Eikwe, Ghana; c Yale School of Nursing, Yale University, New Haven, CT, USA; d Department of Nursing and Midwifery, School of Human and Health Sciences, University of Huddersfield, England, UK

**Keywords:** Type 2 diabetes, obesity, overweight, systematic review, meta-analysis

## Abstract

**Background:**

Type 2 diabetes and obesity are serious public health concerns globally and a growing burden in Africa. Both conditions have serious repercussions on health when they co-occur, yet the extent of their co-occurrence in Africa remains unknown. Therefore, this review aimed to identify the prevalence and associated factors of overweight and obesity among persons with type 2 diabetes in Africa.

**Method:**

A systematic search was conducted on PubMed, MEDLINE, Embase, African Index Medicus (AIM), and African Journals Online (AJOL) for observational studies that reported the prevalence of overweight and/or obesity among type 2 diabetes patients in Africa. The prevalence data from individual studies were aggregated through a random-effects meta-analysis. The *I*^2^ statistic was used to evaluate between-studies heterogeneity, while subgroup analysis and mixed-effects meta-regression were performed to identify sources of heterogeneity. We assessed publication bias using funnel plots and Egger’s test. This review adhered to the Preferred Reporting Items for Systematic Review and Meta-Analysis (PRISMA) guidelines.

**Results:**

Of 1753 records retrieved, 80 articles were eligible for this review, with 74 cross-sectional studies included in the meta-analysis. The pooled prevalence of overweight and obesity was 35.6% and 25.6% respectively, while the overall prevalence of both overweight and obesity was 61.4%. Also, the pooled prevalence of both overweight and obesity across the five geographical areas in Africa ranged from 56.9% in East Africa to 88.5% in Southern Africa. Nineteen factors were significantly associated with overweight and obesity among patients with type 2 diabetes.

**Conclusion:**

The high prevalence of overweight and obesity among patients with type 2 diabetes is a significant public health concern that transcends geographical boundaries within Africa. The findings from this review highlight the need for innovative weight management interventions that are tailored to the cultural context of the African setting.

**KEY MESSAGES**
- There was a high prevalence of overweight and obesity among the type 2 diabetes patients.
- Nineteen factors were identified to be significantly associated with overweight and obesity among type 2 diabetes patients.
- Only 12 out of the 80 included studies primarily focused on the prevalence of overweight and/or obesity which reflects a dearth of interest in this topic.

## Introduction

Obesity and diabetes are two chronic diseases that have emerged as major public health concerns globally, with particular relevance to the African continent. The World Health Organization (WHO) predicts that the prevalence of obesity in Africa is set to increase dramatically, with projections indicating that one in five adults will have obesity by December 2023 [1]. Similarly, Africa is expected to experience a significant burden from the global diabetes epidemic with current projections predicting a 138% in diabetes prevalence by 2024 [2]. Type 2 diabetes (T2DM) is the most prevalent form of diabetes in Africa, accounting for 90% of all diagnosed cases [3].

The relationship between obesity and T2DM has been well-established in the scientific literature with the term ‘diabesity’ used as a distinct phenomenon to highlight their bidirectional relationship [4,5]. Obesity is widely recognized as a major risk factor for the development of T2DM, as excess body fat, particularly around the abdominal area, can lead to insulin resistance; a cardinal feature of T2DM [6]. This pathological pathway is mediated by various mechanisms such as ectopic fat deposition, excess production of adipokines and meta-inflammation [6]. Conversely, the presence of T2DM can also increase the risk of weight gain, partly due to the intake of excess calories by persons with T2DM to compensate for increased energy demands owing to insulin resistance. The co-occurrence of T2DM and obesity has substantial ramifications for health, including the reduction of quality of life [7,8], as well as increasing the risk for cardiovascular diseases [9]. Obesity can have a detrimental impact on the health outcomes of individuals with T2DM, as studies have demonstrated that obesity is associated with higher HbA1c levels (indicating poor glycemic control) and diabetes-related complications [10]. For this reason, the American Diabetes Association (ADA) recommends a 5% reduction in weight for persons with T2DM who have obesity or overweight in order to ensure optimal health [11].

Given the increasing prevalence of diabetes in Africa, it is important to fully understand the health state of persons living with the condition. As highlighted by the ADA, the weight status of T2DM patients is an important indicator of their health. However, to date, no review has systematically synthesized the prevalence of obesity and overweight among persons with T2DM in Africa. In order to address this gap in the literature, this review aimed to identify the prevalence of overweight and obesity among persons with T2DM in Africa by synthesizing the results of relevant studies across the continent. The findings of this review have significant implications for the development of interventions and policies aimed at addressing obesity and T2DM in Africa.

## Methods

This review was conducted in accordance with the Preferred Reporting Items for Systematic Review and Meta-Analysis (PRISMA) 2009 guidelines. The protocol for this review was prospectively registered on PROSPERO (CRD42022348729) prior to the initiation of the study.

### Inclusion and exclusion criteria

The following criteria were used for the included studies: (1) observational studies (cross-sectional, cohort, and case-control); (2) studies conducted in an African country; (3) type 2 diabetes patients; (4) body mass index (BMI) stratified into overweight and obesity according to the World Health Organization (WHO) standards – overweight (25–29.9 kg/m^2^) and obesity (≥30 kg/m^2^) [12]; and (5) articles published between 2000 and 2022. Studies that primarily focused on the prevalence of overweight and obesity among T2DM patients were of utmost priority for inclusion. However, studies that did not primarily focus on our outcome of interest but presented data on overweight and obesity and met the other inclusion criteria were added to this review. With respect to the factors associated with overweight and obesity, studies that identified a significant association with an adjusted effect measure and provided the corresponding 95% confidence interval (CI) were included in this review.

The exclusion criteria were (1) review articles; (2) articles published in languages other than English; (3) studies on both type 1 and type 2 diabetes patients with overweight and/or obesity findings not reported separately for T2DM patients.

### Search strategy

A systematic literature search was conducted on 2 August 2022 to identify studies investigating the prevalence of obesity and overweight in relation to Type 2 diabetes mellitus (T2DM) in Africa. The search was limited to studies published after the year 2000, in order to ensure that the most up-to-date and relevant studies were included. The databases used for the search were PubMed, MEDLINE (*via* Ovid), Embase (*via* Ovid), African Index Medicus (AIM), and African Journals Online (AJOL). The search strategy was guided by the CoCoPop framework; a widely used strategy for asking questions in studies of disease prevalence or incidence [13]. The framework consists of three key domains, the (Co) condition, (Co) context and (Pop) population of study which were framed as (‘obesity’, ‘overweight’), (‘Africa’), and (‘type 2 diabetes mellitus’) respectively. The term ‘prevalence’ was also included in the search strategy. Medical Subject Headings (MeSH) were blended with the free texts to balance the sensitivity and specificity of the search strategy. Boolean combinations (AND, OR, NOT) of search terms were also applied. In addition to the electronic search, the reference lists of the retrieved articles were systematically reviewed to identify any additional relevant studies. The full details of the search strategy are provided in Appendix 1.

### Screening and selection of studies

The screening and selection of studies were conducted in a systematic manner to ensure the inclusion of relevant and high-quality studies in this review. The process consisted of the following steps:

1. Duplicate articles were removed by importing the retrieved articles into EndNote 20.
2. The remaining articles were uploaded to Rayyan (https://www.rayyan.ai/), for title and abstract screening. Articles from AJOL were screened manually as they could not be uploaded directly to Rayyan.
3. The titles and abstracts of the articles were reviewed to identify those that met the inclusion and exclusion criteria for this review. Articles that did not meet these criteria were discarded.
4. The full texts of the remaining articles were thoroughly reviewed to confirm their eligibility for inclusion in the review.

Two reviewers (EE and SA) independently conducted the screening process and disagreements were resolved by consulting the third author (PAD). This approach ensured that the studies included in the review met the predetermined inclusion and exclusion criteria, and that the process was conducted in a thorough and transparent manner.

### Data extraction

A pre-designed and standardized data extraction form was used to systematically retrieve relevant information from each study, including the first author’s name, year of publication, study setting, sample characteristics, study design, and data on overweight and obesity prevalence and associated factors. To ensure the consistency and accuracy of the data extraction process, the form was piloted on a sample of 20 studies prior to its use in the main study. Additionally, to further ensure the quality and reliability of the data, two independent reviewers (EE and SA) performed the data extraction process, and any discrepancies were resolved through consensus.

### Quality assessment

Two independent reviewers (EE and SA) assessed the methodological quality of studies to be included in the analysis. The quality-weighing approach was adopted in the quality assessment by utilizing the quality appraisal checklist designed by the Joanna Briggs Institute (JBI) for cross-sectional studies, case-control studies, and cohort studies. Studies that had 50% or more ‘Yes’ across the quality assessment parameters were considered low risk.

### Data analysis

In this meta-analysis, we employed the meta package in R statistical software to investigate the prevalence of overweight and obesity among persons with T2DM in Africa. To ensure the appropriateness of our analysis, we only included cross-sectional studies in the meta-analysis [14]. Given the potential for considerable variability among the studies included, we utilized the random-effects model. We used the *I*^2^ statistic to quantify the proportion of variability due to heterogeneity across studies, with values of 25%, 50%, and 75% representing low, moderate, and high levels of heterogeneity respectively [15]. To estimate the pooled prevalence, we employed a generalized linear mixed model with the logit transformation, as recommended by Warton and Hui [16]. Additionally, we calculated the 95% confidence interval (CI) for individual studies and the pooled prevalence using the Clopper-Pearson interval.

In addition to the overall analysis, a subgroup analysis was performed to evaluate differences in the prevalence of overweight and obesity among studies stratified by geographical area and gender. Furthermore, we conducted meta-regressions using a mixed-effects model to investigate sources of heterogeneity among studies. The independent variables considered in the meta-regressions included the primary focus of the study on obesity/overweight (yes vs no), study setting (single site vs multisite), BMI assessment (measured vs self-reported), mean time since diagnosis, mean age, publication year, geographical region (east vs west vs central vs north vs south), male-to-female ratio, sampling strategy (probability vs non-probability), and total sample size. Funnel plots and Egger’s test were used to assess for publication bias [17].

## Results

### Search results

**Figure 1.**
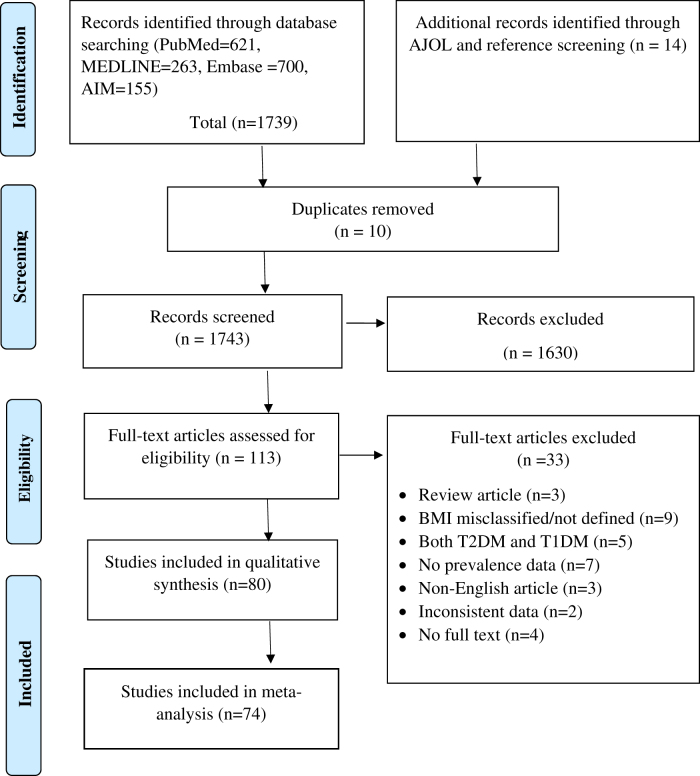
PRISMA flow chart.

The initial literature search for this review was conducted across four databases (PubMed, Embase, MEDLINE, and AIM), yielding a total of 1,739 records. An additional 14 records were identified through AJOL and manual searches of reference list. Following the removal of duplicate records, a total of 1,743 articles were screened based on their titles and abstracts. From this pool, 113 articles were eligible for full-text assessment, with 80 ultimately meeting the inclusion criteria for this review. The meta-analysis was based on 74 cross-sectional studies that were included in the final analysis. The screening process and reasons for exclusion of articles at each stage are clearly outlined in Figure 1, providing a transparent and comprehensive overview of the literature selection process.

### Characteristics of included studies

The current review synthesizes the findings of cross-sectional studies (*n* = 74), case-control studies (*n* = 5), and one cohort study, published between 2004 and 2022. The studies had a combined sample size of 43,631 patients with T2DM. The majority (59.9%) of participants were females. Except for one study [18], all the included studies recruited participants from healthcare facilities. The participants were recruited from 18 distinct countries across the five geographical regions of Africa: East (Kenya, Eritrea, Ethiopia, Eastern Sudan, Sudan, Tanzania, and Uganda), North (Algeria, Egypt, Libya, Morocco, and Tunisia), West (Ghana, Guinea, and Nigeria), South (Botswana, and South Africa), and Central (Cameroon). The majority (47.5%) of the studies were conducted in East Africa, followed by West Africa (32.5%).

Out of the total studies included in the review, only 12 articles specifically focused on the prevalence of overweight and/or obesity among T2DM patients [18–29]. The remaining articles provided data on overweight and/or obesity as an anthropometric or clinical characteristic of the T2DM patients [30–97]. The mean age, diabetes duration, and body mass index (BMI) of the participants varied across studies, with a range of 38.75–66.73 years, 4.0–15.0 years, and 22.24–33.6 kg/m^2^, respectively. Of the 41 studies that measured the mean BMI, 36 (88%) found that T2DM patients had a mean BMI range of 25 kg/m^2^ and above. A summary of the characteristics of the included studies is provided in Table 1.

**Table 1.** Characteristics of the included studies.

First author (year)	Country	Study design	Overweight/ obesity as primary focus	Sample size	Female sample	Mean age ± SD	Mean T2DM duration ± SD	Mean BMI ± SD	Overweight prevalence	Obesity prevalence	Both overweight and obesity prevalence
[31] Sinamaw (2022)	Ethiopia	Cross-sectional	No	258	125	56.7 ± 12.7	6.6 ± 5.24	25.8 ± 4	NR	NR	48.4
[32] Bideberi (2022)	Tanzania	Cross-sectional	No	395	241	58.1 ± 10.3	10.2 ± 7.6	NR	44.8	33.9	78.7
[33] Ebrahim (2022)	Ethiopia	Comparative cross-sectional	No	120	56	38.75 ± 10.58	NR	NR	16.7	NR	NA
[20] Bizuayehu (2022)	Ethiopia	Cross-sectional	Yes	314	103	NR	NR	NR	36.3	18.8	55.1
[34] Seid (2022)	Ethiopia	Cross-sectional	No	322	147	52	NR	NR	37.6	11.5	49.1
[35] Abebe (2022)	Ethiopia	Prospective observational study (cross-sectional)	No	138	56	49.35 ± 11.58	6.21 ± 4.34	26.24 ± 3.22	36.2	21.7	57.9
[36] Abera (2022)	Ethiopia	Cross-sectional	No	325	186	54.0	9.0	NR	19.1	3.7	22.8
[37] Umelo (2022)	Nigeria	Cross-sectional	No	108	64	55.37 ± 11.10	9.37 ± 7.28	26.66 ± 4.23	35.2	21.3	56.5
[38] Junaid (2022)	Nigeria	Cross-sectional Comparative	No	96	48	66.73 ± 5.18	NR	NR	42.7	15.6	58.3
[39] Yusuf (2022)	Nigeria	Cross-sectional	No	274	194	60.0 ± 9.8	NR	NR	36.1	27.7	63.8
[40] Sebai (2022)	Tunisia	Cross-sectional	No	457	255	56.5 ± 9.2	8.62 ± 6.52	31.0 ± 7.2	NR	49.2	NA
[41] Kebede (2021)	Ethiopia	Cross-sectional	No	327	150	53 + 17	NR	26 + 3.8	56.0	18.0	74.0
[19] Abdissa (2021)	Ethiopia	Cross-sectional	Yes	334	152	51.42 ± 13.33	6.95 ± 5.37	NR	29.3	6.9	36.2
[42] Shigidi (2021)	Sudan	Case-control	No	736	316	58.5 ± 12.5	NR	NR	NR	5.6	NA
[43] Ibrahim (2021)	Nigeria	Retrospective cross-sectional	No	300	174	61.9 ± 11.8	NR	26.2 ± 4.9	34.0	18.0	52.0
[44] Yosef (2021)	Ethiopia	Cross-sectional	No	245	110	48.6 ± 14.9	NR	24.95 ± 3.99	35.1	19.8	54.9
[45] Omar (2021)	Eastern Sudan	Cross-sectional	No	350	205	NR	NR	25.9 (5.8)	36.6	22.9	59.5
[46] Regassa (2021)	Ethiopia	Retrospective Cohort study	No	458	200	NR	NR	NR	NR	13.32	NA
[47] Djonor (2021)	Ghana	Cross-sectional	No	271	194	56.6 ± 13.8	NR	28.64 ± 3.97	46.1	34.5	80.6
[48] Kotiso (2021)	Ethiopia	Case-control	No	386	198	NR	NR	NR	37.8	18.4	56.2
[49] Saasita (2021)	Uganda	Cross-sectional	No	206	147	NR	NR	NR	38.8	26.2	65.0
[50] Chetoui (2020)	Morocco	Cross-sectional	No	1456	1068	56.16/±11.76	8.63 ± 6.8	NR	42.1	26.9	69.0
[21] Tino (2020)	Uganda	Retrospective Chart review (cross-sectional)	Yes	1275	770	NR	NR	NR	36.0	27.0	63.0
[51] Otieno (2020)	Kenya	Cross-sectional	No	385	252	63.3	NR	26.7 (4.6)	40.0	22.3	62.3
[52] Zerga (2020)	Ethiopia	Cross-sectional	No	330	160	NR	NR	NR	26.4	11.2	37.6
[53] Munyogwa (2020)	Tanzania	Cross-sectional	No	330	189	40.27 ± 13.31	NR	NR	NR	93.3	NA
[54] Taderegew (2020)	Ethiopia	Cross-sectional	No	422	229	54.16 ± 10.61	6.2 ± 4.6	23.28 ± 3.64	18.5	13.3	31.8
[55] Haile (2020)	Ethiopia	Cross-sectional	No	248	129	49.6 ± 13.3	NR	26.5 ± 3.8	49.2	21.4	70.6
[56] Akalu (2020)	Ethiopia	Cross-sectional	No	280	108	61.2 ± 7.3	NR	NR	73.2	20.4	93.6
[57] Kouitcheu (2020)	Cameroon	Comparative Cross-sectional	No	93	61	54.70 ± 1.07	NR	26.25 ± 0.2741	NR	NR	77.4
[58] Achila (2020)	Eritrea	Descriptive cross-sectional	No	309	145	57.8 ± 11.5	12.1 ± 7.4	24.6 ± 4.4	35.0	9.4	44.4
[59] Abdissa (2020)	Ethiopia	Cross-sectional	No	366	163	50.1 ± 14.28	NR	NR	23.0	5.7	28.7
[60] Abdallah (2019)	Egypt	Comparative cross-sectional	No	100	44	NR	NR	NR	62.0	22.0	84.0
[61] Bello-Ovosi (2019)	Nigeria	Cross-sectional	No	322	161	53.5 ± 10.8	NR	27.8 ± 6.4	32.4	32.7	65.1
[62] Karau (2019)	Kenya	Cross-sectional	No	151	105	58.2 ± 12.2	9.2 ± 6.9	28.53	41.1	38.4	79.5
[63] Anioke (2019)	Nigeria	Cross-sectional	No	140	63	NR	NR	NR	55.0	35.7	90.7
[22] Kiros (2019)	Ethiopia	Cross-sectional	Yes	365	167	NR	NR	NR	NR	NR	40.8 M 38.4 F 43.7
[64] Asamoah-Boaheng (2019)	Ghana	Retrospective review of medical records (cross-sectional)	No	15271	9569	M 60.6 ± 13.0 F 61.6 ± 13.1	NR	NR	27.6	14.6	42.7
[27] Gezawa (2019)	Nigeria	Cross-sectional	Yes	220	123	NR	NR	27.2 (6.4)	35.5	27.4	62.9
[65] Ekoru (2019)	Ghana, Kenya, Nigeria	Retrospective case control	No	2784	1698	56 ± 11	NR	NR	36.0	27.0	63.0
[66] Bekele (2019)	Ethiopia	Cross-sectional	No	374	198	56.3 ± 11.5	NR	NR	28.9	12.8	41.7
[67] Fekadu (2019)	Ethiopia	Cross-sectional	No	228	110	43 ± 12.4	NR	22.24 ± 5.12	33.2	6.3	39.5
[28] Kasimu (2019)	Nigeria	Retrospective review of medical records (cross-sectional)	Yes	182	116	53.2 ± 16.9	NR	30.8 ± 6.5	23.6	57.7	81.3
[30] Shiriyedeve (2019)	Botswana	Cross-sectional	No	170	112	51.0 ± 13.3	NR	NR	26.9	58.1	85
[68] Bouhajja (2018)	Tunisia	Cross-sectional	No	108	60	56.49 ± 10.19	NR	33.30	NR	51.9 M 37.5 F 63.3	NA
[69] Sarfo-Kantanka (2018)	Ghana	Cross-sectional	No	780	450	57.4 ± 9.4	9.8 ± 5.6	27.5	NR	33.6 M 27.9 F 37.8	NA
[70] Wolde (2018)	Ethiopia	Retrospective follow-up study (cross-sectional)	No	341	196	51.7 (SD ± 11.5)	NR	NR	28.7	13.2	41.9
[25] Balogun (2018)	Nigeria	Cross-sectional	Yes	709	378	51.9 ± 13.9	NR	26.6 ± 5.2	23.1	35.7	58.8
[71] Birkinshaw (2018)	South Africa	Cross-sectional	No	50	26	57.9	7.0	NR	22.0	66.0	88.0
[23] Damian (2017)	Tanzania	Cross-sectional	Yes	227	154	56 (±11.9)	NR	NR	44.9	40.1	85.0
[72] Gudjinu (2017)	Ghana	Unmatched case control	No	48	27	NR	NR	NR	33.3	33.3	66.6
[73] Otieno (2017)	Kenya	Descriptive cross-sectional	No	220	131	57.1 ± 8.6	NR	NR	36.8	24.5	61.3
[74] Ojieabu (2017)	Nigeria	Retrospective study of case file (cross-sectional)	No	167	103	63.9 ± 9.6	NR	NR	32.3	22.2	54.5
[18] Ali (2017)	Sudan	Descriptive cross-sectional	Yes	1337	773	NR	NR	NR	39.9	24.5	64.4
[75] Mwanri (2017)	Tanzania	Cross-sectional	No	119	91	57.7 ± 12.8	NR	26.0 ± 4.3	42.9	15.1	58.0
[76] Goie (2016)	South Africa	Descriptive cross-sectional	No	280	201	59 ± 9.28	NR	NR	25.0	66.0	91.0
[77] Habtewold (2016)	Ethiopia	Cross-sectional	No	264	140	55.9 ± 10.9	NR	NR	37.1	12.9	50.0
[29] Mogre (2016)	Ghana	Cross-sectional	Yes	378	246	47.3 ± 12.73	5.0 ± 3.5	26.8 ± 5.7	38.6	20.1	58.7
[78] Belkacemia (2016)	Algeria	Cross-sectional	No	180	107	62.56 ± 8.1	5.0	27.15	42.8	18.3	61.1
[79] Adebola (2016)	Nigeria	Comparative cross-sectional	No	97	53	58.9 ± 14.95	7.6 ± 6.4	NR	27.8	52.6	80.4
[80] Diaf (2015)	Algeria	Cross-sectional	No	238	152	57.36 ± 11.93	6.8 ± 3.7	27.89 ± 4.58	38.6	31.5	70.1
[24] Adeniyi (2015)	South Africa	Cross-sectional	Yes	327	230	NR	NR	NR	31.8	60.2	92.0
[81] Camara (2015)	Cameroon Guinea	Cross-sectional	No	1267	775	58.4 ± 10.5	7.6 ± 6.3	27.4 ± 5.8	NR	NR	64.5
[82] Nadge (2014)	Kenya	Cross-sectional	No	218	122	56.6 ± 9.3	10.7 ± 6.6	NR	61.0	18.0	79.0
[83] Kamuhabwa (2014)	Tanzania	Cross-sectional	No	469	298	54.94 ± 11.93	7.19 ± 6.04	27.06 ± 5.34	32.5	26.9	59.4
[84] Mogre (2014)	Ghana	Cross-sectional	No	200	154	56.2 ± 12.13	5.23 ± 5.00	23.86 ± 4.64	NR	NR	32.0
[85] Brenyah (2013)	Ghana	Cross-sectional	No	350	251	54.9 ± 11.0	5.8 ± 4.8	25.5 ± 4.4	40.5	14.7	55.2
[86] Okafor (2012)	Nigeria	Cross-sectional survey	No	233	135	55.7 ± 11.7	6.7 ± 6.3	27.0 ± 6.2	NR	NR	60.1
[87] Berraho (2012)	Morocco	Cross-sectional	No	525	361	NR	NR	NR	42.7	31.2	73.9
[88] Danquah (2012)	Ghana	Case-control	No	675	504	54.7 ± 13.4	NR	25.9 ± 5.1	53.0	19.0	72.0
[89] Acquah (2011)	Ghana	Cross-sectional	No	79	57	M 60.00 ± 9.12 F 55.63 ± 12.28	NR	M 24.46 ± 3.51 F 30.00 ± 6.87	NR	NR	43.0
[90] Baba (2010)	Nigeria	Cross-sectional	No	75	45	M 58.3 ± 10.3 F 57.2 ± 9.4	NR	26.0 ± 5.1	36.0	16.0	52.0
[91] Elnasri (2008)	Sudan	Cross-sectional	No	250	155	52.0 ± 13	NR	28.7 ± 6.4	29.6	25.6	55.2
[92] Alshkri (2008)	Libya	Cross-sectional pilot study	No	99	61	56.0 ± 9.5	9.4 ± 7.8	33.6 ± 5.6	NR	74.4	NA
[93] Ajayi (2010)	Nigeria	Retrospective review of medical records (cross sectional)	No	308	183	60.90 ± 11.60	NR	25.47 ± 4.55	36.5	12.2	48.7
[94] Choukem (2007)	Cameroon	Cross-sectional	No	191	98	59.7 ± 9.4	6.2 ± 0.4	25.4 ± 5.4	NR	NR	48.5
[95] Akande (2007)	Nigeria	Cross-sectional	No	121	77	57.3 ± 10	NR	NR	38.9	25.6	64.5
[26] Fasanmade (2007)	Nigeria	Cross sectional	Yes	258	135	NR	NR	M 25.6 F 27.1	39.9	18.6	58.5
[96] Adediran (2007)	Nigeria	Cross-sectional	No	113	70	M 62.1 ± 12.2 F 60 ± 11.5	M 3.71 ± 2.3 F 3.92 ± 3.6	M 26 ± 6.1 F 27.32 ± 5.7	28.3	20.4	48.7
[97] Mkuyana (2004)	Zimbabwe	Cross-sectional	No	109	77	55.0 ± 9	M 4.2 ± 4.0 F 5.3 + 4.5	M 25.7 ± 4.6 F 28.2 ± 6.2	NR	32.0	NA

SD: Standard deviation; BMI: Body Mass Index; NR: not reported; NA: Not available; M: Male; F: Female.

### Quality of included studies

All the included studies had a quality score above 50% with a mean score of 72%. Two authors (EE and SA) agreed on almost 90% of the methodological quality of the studies after the independent assessment. Disagreements were discussed and consensus was attained after consulting the third author (PAD). The result of the quality assessment is provided as a supplemental file [Appendix 2].

### Prevalence of overweight

**Figure 2.**
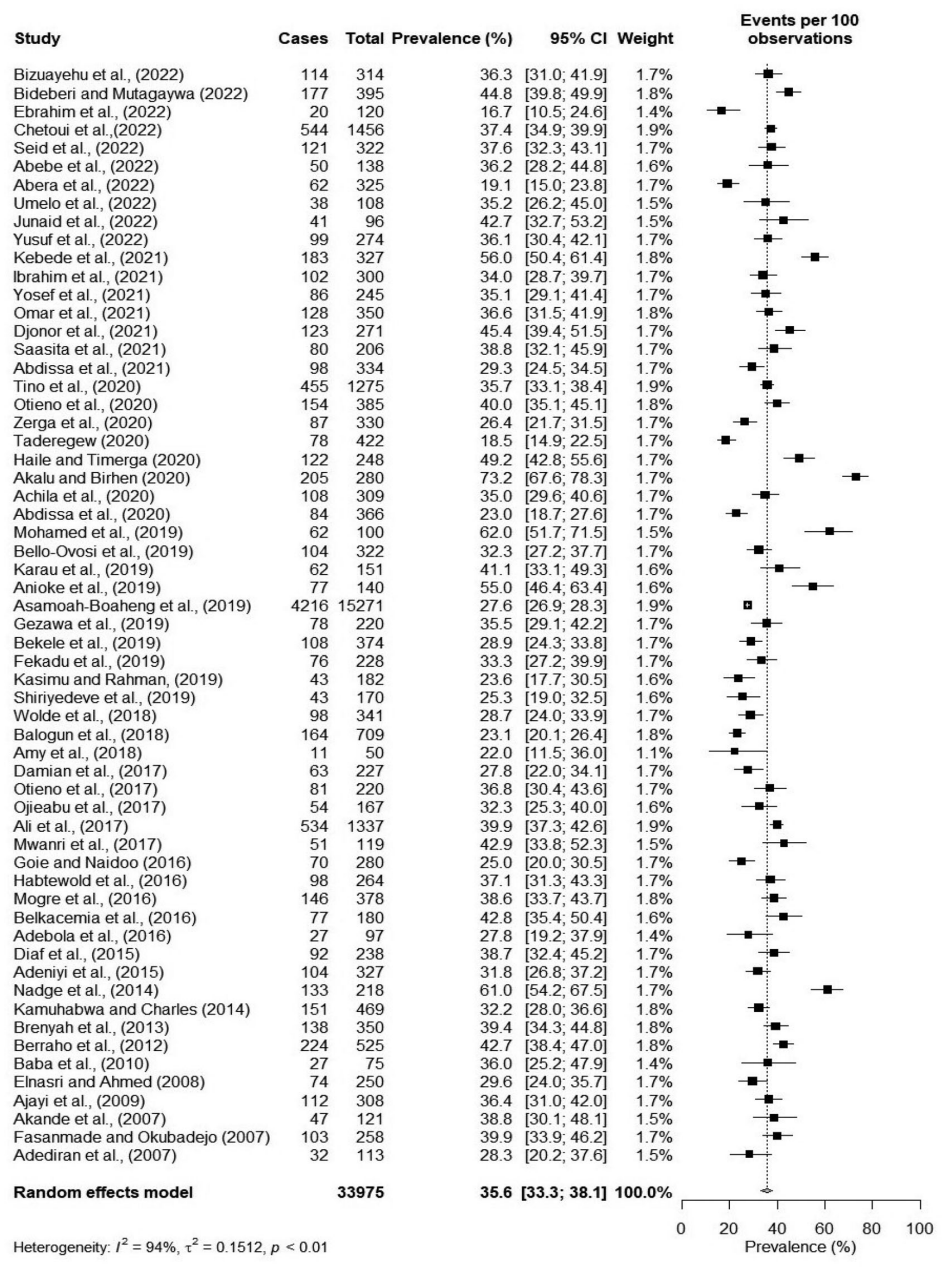
Forest plot of the pooled prevalence of overweight among type 2 diabetes patients in Africa.

Sixty cross-sectional studies provided data on the prevalence of overweight among persons with T2DM. The prevalence of overweight ranged from 16.7% [33] to 73.2% [56]. The pooled prevalence of overweight was 35.6% (95% CI: 33.3–38.1). There was a significantly high heterogeneity among the studies (*I*^2^ = 94%, *p* < 0.01) as shown in Figure 2.

### Prevalence of obesity

**Figure 3.**
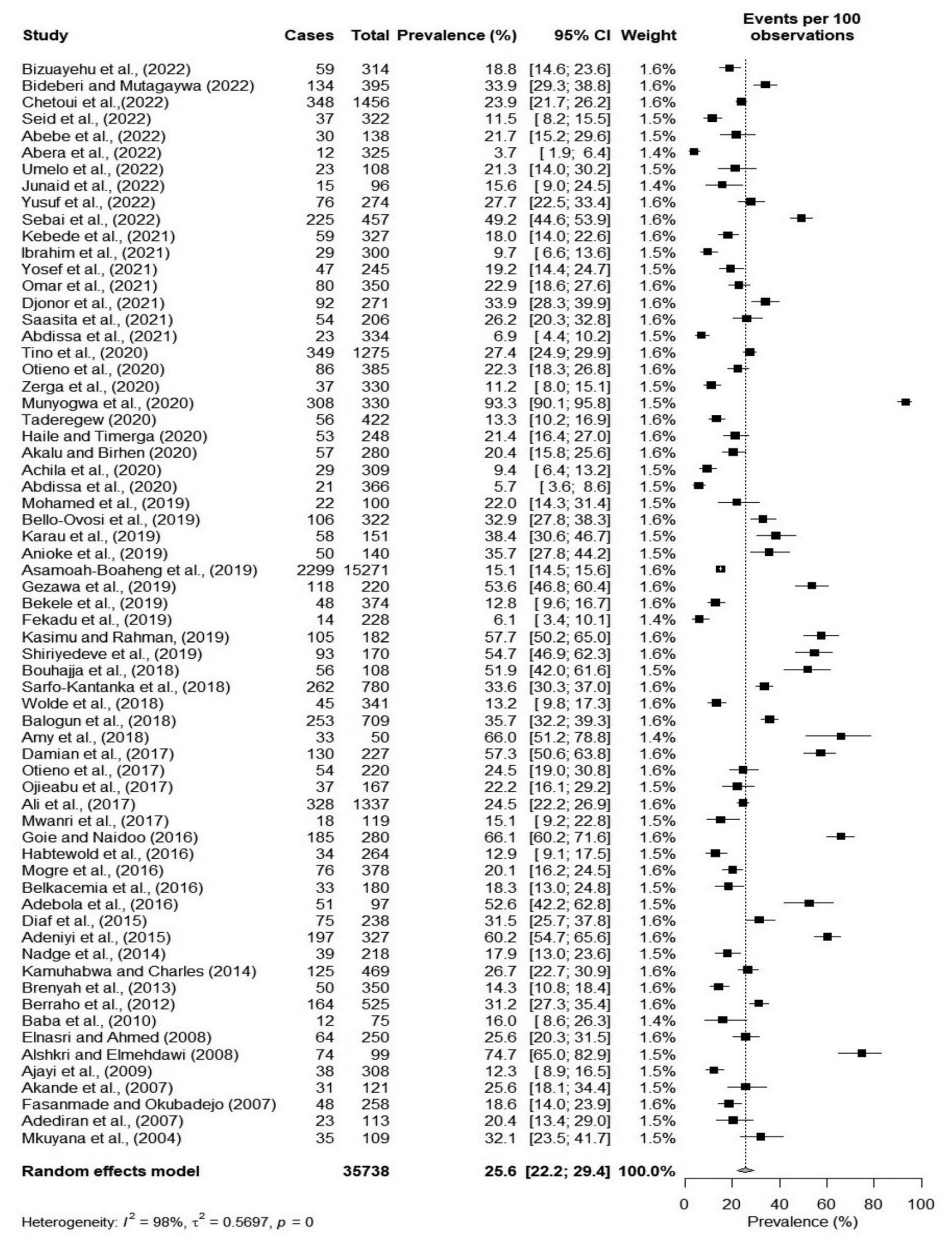
Forest plot of the pooled prevalence of obesity among type 2 diabetes patients in Africa.

The prevalence of obesity as reported by sixty-eight cross-sectional studies ranged from 3.7% [36] to 93.3% [53]. The pooled prevalence of obesity was 25.6% (95% CI: 22.2–29.4). There was a significantly high heterogeneity among studies (*I*^2^ = 98%, *p* < 0.001) as shown in Figure 3.

### Prevalence of overweight and obesity

**Figure 4.**
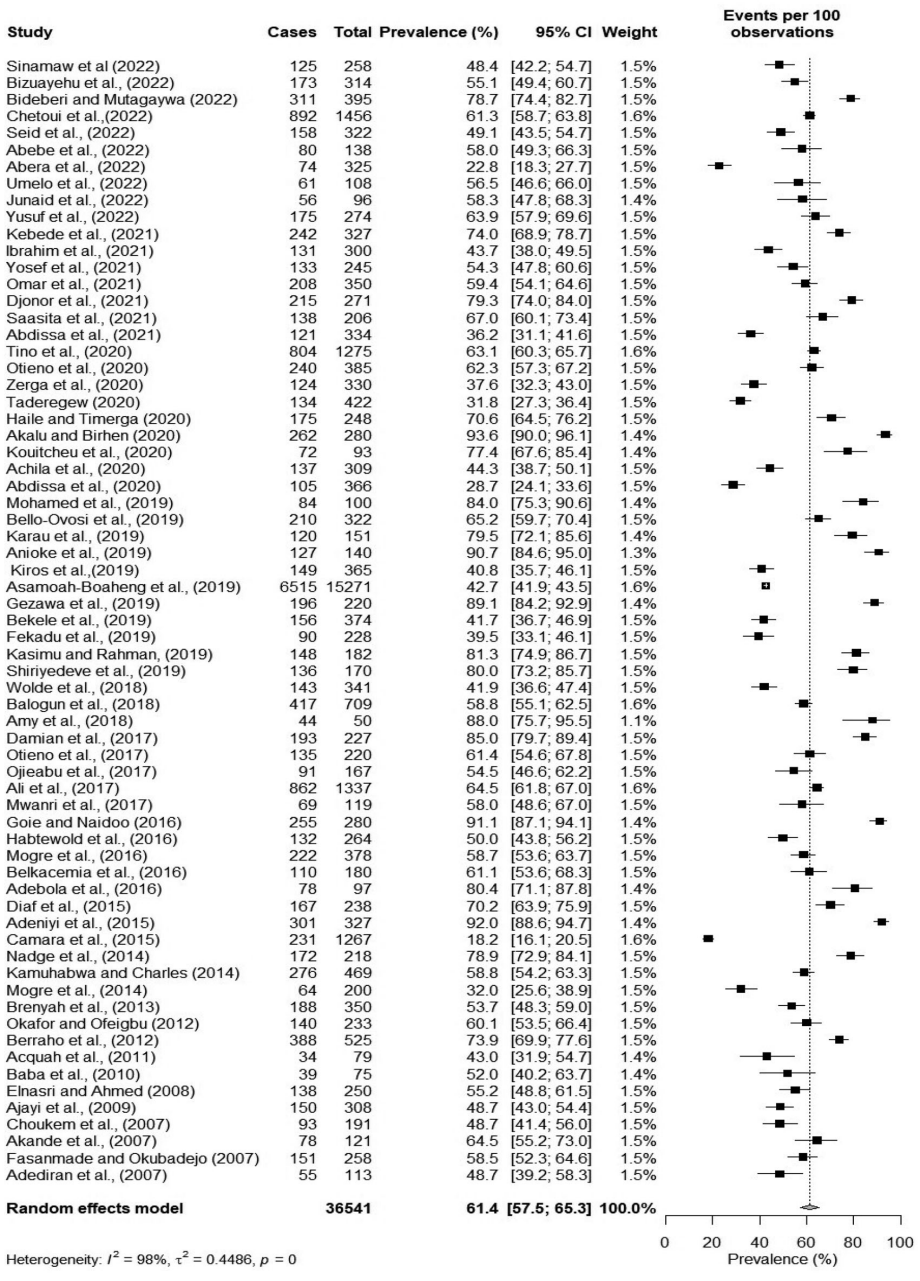
Forest plot of the pooled prevalence of both overweight and obesity among type 2 diabetes patients in Africa.

Sixty-nine cross-sectional studies provided data on both overweight and obesity. Of these, only seven studies presented results for both overweight and obesity as a single entity. The prevalence of both overweight and obesity from the remaining studies was calculated by simply summing up the separately reported prevalence data of overweight and obesity. The prevalence of both overweight and obesity ranged from 22.8% [36] to 93.6% [56]. The pooled prevalence of both overweight and obesity was 61.4% (95% CI: 57.5–65.3). There was a significantly high heterogeneity among the studies (*I*^2^ = 98%, *p* < 0.001) (Figure 4).

### Subgroup and meta-regression analysis

The individual studies were stratified according to the five geographical regions in Africa and their respective overweight and obesity prevalence were pooled. The prevalence of overweight and obesity was highest (88.5% [95% CI: 81.4–93.1]) in Southern Africa as compared to 70.0% in North Africa, 64.0% in Central Africa, 61.4% in West Africa, and 56.9% in East Africa. The studies from West and East Africa showed a significant level of heterogeneity (*I*^2^ =97%, *p* < 0.01). In terms of gender-based comparisons, the prevalence of overweight and obesity was 65.8% (95% CI: 59.1–71.8) among females and 50.1% (95% CI: 42.7–57.4) among males. Also, females had 89% more odds of being overweight and obese compared to their male counterparts [OR 1.89 (95% CI: 1.62–2.21)].

The results of the meta-regression analyses demonstrated that geographical regions and sampling strategies significantly influenced the combined prevalence of obesity and overweight among individuals withT2DM. Notably, there was a marginal reduction in between-study heterogeneity to 96.6% after accounting for the geographical region where the study was conducted. Studies conducted in Southern Africa (Estimate = 1.76, SE = 0.35, *p* < 0.0001) and North Africa (Estimate = 0.59, SE = 0.30, *p* = 0.048) reported significantly higher prevalence as compared to those from East Africa. Additionally, studies that employed a probability sampling strategy reported a lower prevalence of obesity and overweight compared to those that utilized a non-probability sampling strategy (Estimate = −0.72, SE = 0.22, *p* = 0.0013). A summary of the meta-regression of the prevalence of overweight and obesity among T2DM patients can be found in Appendix 3.

### Publication bias

There was an asymmetrical distribution of the funnel plot for overweight, obesity, and both overweight and obesity which implies a publication bias among studies. Statistical evidence of publication bias was further confirmed with Egger’s test with *p*-value of 0.0010, 0.0120 and <0.001 for overweight, obesity, and both overweight and obesity respectively. The funnel plot for overweight, obesity, and both overweight and obesity and results from Egger’s test can be found in Appendix 4.

### Factors associated with overweight and obesity

Six studies met the eligibility criteria for this review, and all provided adjusted effect measures in the form of odds ratio (OR) [19–22,24,29]. A total of 19 factors were identified as being significantly associated with overweight and obesity among patients with T2DM. The factors are grouped under three categories: sociodemographic factors (gender, age, place of residence, employment, education level, average monthly income/economic status, and family history of overweight and obesity); behavioral factors (smoking history, alcohol intake, physical activity/walk/exercise, attempt to lose weight through exercise, weight management plan, diet, and counselling for weight reduction); and clinical factors (triglycerides, T2DM comorbidity/complication, abdominal/central obesity, T2DM duration, and T2DM treatment). A summary of all the factors associated with overweight and obesity is presented in Table 2.

**Table 2.** Summary of the factors associated with overweight and obesity among T2DM patients in Africa.

*N* studies	Author	Factors	Significant responds	Adjusted results OR (95% CI)
		Sociodemographic		
3	[21] Tino [20] Bizuayehu [24] Adeniyi	Gender	Male Female Female	0.45 (0.340–0.593) 3.0 (1.6–5.7) 2.5 (1.5–4.3)
2	[19] Abdissa [22] Kiros	Place of residence	Urban Urban	1.8 (1–3) 3.4 (1.26–9.4)
2	[19] Abdissa [21] Tino	Economic status	AMI ≥58.9 USD Middle income Upper income	3.4 (1.8–6.7) 1.83 (1.320–2.550) 2.10 (1.452–2.994)
1	[24] Adeniyi	Employment	Unemployed	2.3 (1.2–4.5)
1	[21] Tino	Age	65+	0.52 (0.353 to 0.770)
1	[24] Adeniyi	Education	Secondary Tertiary	3.7 (1.8–7.6) 6.2 (1.7–23.2)
1	[19] Abdissa	Family history of overweight or obesity	Positive family history	1.9 (1.1–3.4)
		Behavioral		
1	[24] Adeniyi	Smoking	Yes	3.5 (1.4–8.9)
1	[22] Kiros	Alcohol intake	Yes	2.9 (1.5–5.5)
2	[19] Abdissa [22] Kiros	Physical activity/exercise	Inactive Inactive	2.1 (1.2–3.5) 4 (1.19–13.8)
1	[29] Mogre	Attempt to lose weight through exercise	Yes	2.4 (1.2–4.9)
1	[29] Mogre	Weight management plan	Yes	3.1 (1.6–6.2)
1	[22] Kiros [21] Tino	Diet	Poor dietary intake Rare intake of fruits and vegetables	8 (4.02–15.5) 0.66 (0.475–0.921)
1	[29] Mogre	Counselling for weight reduction	Yes	3.8 (1.9–7.6)
		Clinical		
1	[21] Tino	T2DM treatment	Dual therapy	2.17 (1.024–4.604)
1	[29] Mogre [22] Kiros	T2DM duration	≥5 years 3–6 years	0.5 (0.3–0.9) 2.8 (1, 7.85)
1	[20] Bizuayehu	Triglycerides	≥200 mg/dl	3.6 (1.6–8.3)
	[19] Abdissa [21] Tino	T2DM comorbidity/complication	Hypertension Hypertension Peripheral neuropathy	2.4 (1.4–4) 1.70 (1.264–2.293) 1.40 (1.039–1.834)
2	[22] Kiros [29] Mogre	Abdominal/central obesity	Present Present	3.4 (1.64, 6.91) 5.6 (3.3–9.6)

*N*: Number of studies; AMI: Average monthly income; USD: United States Dollar.

### Sociodemographic factors

Gender, place of residence, and economic status were identified as the most significant sociodemographic factors associated with overweight and obesity in T2DM patients. With respect to gender, the findings of three studies [20,21,24] revealed that females were more likely to be overweight and obese than their male counterparts. The adjusted odds ratios (AOR) for obesity among female T2DM patients ranged from (AOR 2.5 [95% CI: 1.5–4.3]) [24] to (AOR 3.0 [95% CI: 1.6–5.7]) [20].

Two studies also found a significant association between place of residence and overweight and obesity in T2DM patients [19,22]. In both studies, patients living in urban areas had higher odds of being overweight and obese compared to those residing in rural areas. Specifically, Abdissa et al.'s study [19] reported that T2DM patients living in urban areas were almost twice as likely to be overweight and obese (AOR 1.8 [95% CI: 1–3]) while Kiros et al.’s study [22] found that the strength of association was even greater, with T2DM patients in urban areas being more than three times as likely to be overweight and obese (AOR 3.4 [95% CI: 1.26–9.4]).

Two studies identified a significant association between overweight and obesity and economic status or average monthly income (AMI) in T2DM patients [19,21]. Both studies found that the odds of being overweight and obese increased with higher economic status or AMI. One study reported that T2DM patients with higher income were more than twice as likely to be overweight and obese as compared to those with lower income (AOR 2.10 [95% CI: 1.452–2.994]) [21]. The other study reported an even stronger association, with T2DM patients with AMI ≥ 58.9 United States Dollars (USD) having more than three times the odds of being overweight and obese as compared to those with AMI below 58.9 USD (AOR 3.4 [95% CI: 1.8–6.7]) [19].

#### Behavioral factors

Several studies investigated the relationship between physical activity, obesity, and T2DM. The findings suggest that T2DM patients who engaged in less physical activity were more likely to be overweight or obese. For example, Abdissa et al. found that T2DM patients who did not exercise were twice as likely to be obese as compared to those who engaged in active exercise (AOR 2.1 [95% CI: 1.2–3.5]) [19]. Similarly, Kiros et al. found that T2DM patients who engaged less in vigorous activity were four times as likely to be overweight (AOR 4 [95% CI: 1.19–13.8]) [22]. However, Mogre et al. found that T2DM patients who exercised with the intention to lose weight were more than twice as likely to be overweight and obese as compared to those who did not exercise (AOR [2.4 95% CI: 1.2–4.9]) [29]. This finding may be because, although the patients exercised, their level of commitment to other weight reduction modalities was suboptimal. In fact, it is worth noting that in the same study, T2DM patients who had a weight management plan were more likely to be obese.

One study identified a significant association between smoking and obesity with adjusted effect measure. The result revealed that T2DM patients who smoked were more than three times as likely to be obese as compared to those who have never smoked (AOR 3.5 [95% CI 1.4–8.9]) [24]. Similarly, a significant association between overweight/obesity and alcohol intake was identified in only Kiros et al.'s study. The results revealed that T2DM patients who drink alcohol were almost three times likely to be overweight as compared to those who do not drink alcohol (AOR 2.9 [95% CI: 1.5–5.5]) [22].

#### Clinical factors

Results from this review indicate that T2DM-associated comorbidities and complications, such as hypertension and peripheral neuropathy, have a significant association with overweight and obesity. Specifically, the odds of overweight and obesity among T2DM patients with hypertension (referred to as abnormal or high blood pressure in some studies) ranged from (AOR 1.70 [95% CI: 1.264–2.293]) [21] to (AOR 2.4 [95% CI: 1.4–4]) [19]. Furthermore, T2DM patients with peripheral neuropathy were found to be more likely associated with overweight and obesity as compared to those without this complication (AOR 1.40 [95% CI: 1.039–1.834]) [21].

Additionally, two studies found a significant association between overweight and obesity and abdominal/central obesity in two studies [22,29]. Both studies found T2DM patients with abdominal/central obesity to be more likely associated with overweight and obesity, with the strength of association ranging from (AOR 3.4 [95% CI: 1.64–6.91]) [22] to (AOR 5.6 [95% CI: 3.3–9.6]) [29].

Furthermore, Tino et al. [21] explored the association between overweight and obesity and diabetes treatment or therapy. The study found that T2DM patients on dual therapy (two antidiabetic medications) were significantly more likely to be overweight and obese. The authors further discussed that the high odds of overweight and obesity in this population may be attributed to the weight-gaining side effects of certain medications, specifically thiazonlinedione and sulphonylureas when coupled with insulin.

## Discussion

To the best of our knowledge, this systematic review and meta-analysis is the first of its kind to present evidence on the extent of overweight and/or obesity prevalence among persons with T2DM in Africa. This review is also novel in extensively exploring the factors associated with overweight/obesity among persons with T2DM in Africa.

Results from our meta-analysis revealed that the prevalence of overweight and obesity among T2DM patients in Africa was 35.6% and 25.6% respectively, while the overall prevalence of both overweight and obesity was 61.4%. This finding is consistent with the belief that larger body size is commonly perceived as a marker of good health and wealth in Africa, which can act as a barrier to weight reduction practices [98]. In developed countries, higher obesity and overweight prevalence among T2DM patients have been reported. The prevalence rates can be as high as 78% in France [99] and over 85% in the United Kingdom [100] and the United States of America [101]. Given that Africa is projected to be the most urbanized continent by 2025 [102], it is likely that future studies will record a higher prevalence of overweight and obesity in Africa as compared to developed countries. Therefore, it is essential to implement weight reduction interventions in Africa in a timely manner. Generally, there is a lack of strictly enforced policies on nutrition regulations and marketing in most African countries, and this predispose the population to unhealthy eating habits, which in turn increases the risk of overweight and obesity [23,103].

Furthermore, we observed variations in the prevalence of overweight and obesity across the five geographical regions of Africa, with the highest rates observed in Southern Africa (88.5%) and the lowest in East Africa (56.9%). This variation may be attributed to factors such as differences in urbanization transition across the regions, cultural influences, access to healthcare, genetics, and food security.

We found that females were more likely to be overweight and obese compared to their male counterparts. This is partly attributed to their hormonal and body composition, preference for large body size, consumption of foods with high sugar contents, [104] and high food craving score [105]. Also, from the scope of Africa’s socio-cultural practice, females’ dietary plan and physical activity levels are modeled in a manner that increases their weight to make them look attractive to their groom [106]. Additionally, individuals living in urban areas and those with higher economic standing were found to be more likely to be overweight and obese, which may be linked to the adoption of western lifestyles and increased consumption of energy-dense foods [19,107].

The ADA emphasizes the importance of weight management in managing T2DM and recommends adopting a healthy lifestyle, such as regular exercise, increased intake of fruits and vegetables, limited alcohol intake, and avoiding sugar-sweetened beverages [11]. Consistent with the ADA guidelines, we found that T2DM patients who took alcohol, engaged in less physical activities, regularly took soft drinks, and had a poor dietary intake were more likely to be overweight and obese. However, we also found an unexpected association between regular consumption of fruits and vegetables, weight management planning, and weight reduction counseling and overweight/obesity among T2DM patients. This contrasting pattern may be due to poor adherence to weight reduction modalities. It is worth noting that these factors were explored with cross-sectional study design which are inappropriate in establishing causal relationships [14]. This may also contribute to the contrasting patterns observed.

Our findings on the clinical factors associated with overweight and obesity reveal that several elements, including hypertension, central obesity, and high triglyceride levels, are well-established to have a common relationship with these conditions. These are all clinical parameters of metabolic syndrome and are frequently observed among patients with T2DM [108]. The increased risk for hypertension among individuals with obesity is believed to be mediated by the activation of the sympathetic nervous system, increased insulin resistance, and the release of adipokines [109]. Additionally, our study identified that T2DM patients with peripheral neuropathy were more likely to be obese. This is in line with previous reports from outside Africa, which have found that T2DM patients with peripheral neuropathy have higher body mass index (BMI) values [110]. Furthermore, our study revealed that having central obesity was associated with general obesity, which is in agreement with the well-established contemporary relationship between these two conditions.

### Strengths and limitations

The major strength of this review lies in its novelty as it serves as a benchmark for future studies on the same subject in Africa and beyond. Also, the use of only cross-sectional studies in the meta-analysis ensured robustness of the results since cross-sectional studies are most appropriate for prevalence studies. Almost all the included studies recruited the patients from the health facility, thereby ensuring that the overweight or obesity data were measured rather than self-reported. Despite the objectivity of overweight or obesity data from the facility-based studies, the generalization of our results seems tentative since vast majority of type 2 diabetes patients outside the facility were not captured. Moreover, our analysis identified a significant publication bias among the included studies, which may have led to an underestimation of the true effect size of the prevalence of overweight and obesity among T2DM patients in Africa. Future research should aim to control for this bias in order to obtain a more accurate and precise estimate of the true effect size. Although studies from all the five geographical areas in Africa were captured, some areas were highly underrepresented with limited articles. There was much variation in the characteristics of the studies which may have led to the high heterogeneity of the results. Non-English articles, and access-restricted articles were excluded, therefore there is the possibility of missing some vital studies which may have been valuable for this review.

### Recommendations

Considering the serious sequelae associated with obesity and T2DM, more research especially those employing large-scale epidemiological approaches with a representative sample are needed to obtain an in-depth understanding of overweight and obesity among T2DM patients. Nevertheless, our findings present a serious public health concern with implications on the growing burden of chronic diseases in Africa. Hence, a cutting-edge intervention is required to mitigate the existential threat imposed by diabetes and obesity. This intervention should be contextually appropriate to the African setting by considering African traditional values that inform decisions for weight control.

Our findings on the factors associated with overweight and obesity had some patterns of inconsistency with the global literature. This is partly due to the cross-sectional study design used by the included studies in assessing the causal relationship. Future research should therefore utilize more robust designs such as cohort studies and random control trials to explore the factors associated with overweight and obesity among T2DM patients.

Given the likelihood of overweight and obesity prevalence among T2DM patients with weight management plan and weight reduction counselling, the level of adherence to weight management modalities is quite questionable. It is crucial that future research should explore the context of weight management plan, weight reduction counselling, and their effectiveness in overweight and obesity management among T2DM patients in Africa. The level of adherence to weight management modalities should be comprehensively investigated. As a matter of fact, health providers’ interventions towards weight reduction should take an iterative approach to enforce patients’ adherence to weight management plans and thus, help achieve and sustain desired outcomes.

## Conclusion

This review identified a high prevalence of overweight and obesity which transcend across the geographical areas of Africa. Notwithstanding the generality of high prevalence of overweight and obesity, some subsets of T2DM patients such as females and those living in urban areas were more likely to be overweight and obese. Hence, it is imperative that public health interventions should target these subgroups to enhance cost-effectiveness.

## Supplementary Material

Supplemental Material

## Data Availability

All data for this review can be accessed in this manuscript and its supplementary files.
