# Supplementary material for: Prevalence and associated factors of overweight and obesity among persons with type 2 diabetes in Africa: a systematic review and meta-analysis
Source: Ann Med. 2023 Feb 23;55(1):696–713. doi: 10.1080/07853890.2023.2182909 (PMC9970251; doi:10.1080/07853890.2023.2182909)
Supplement: Supplemental Material [file IANN_A_2182909_SM3068.docx]

**Appendix 1.** Search strategy used for the electronic databases

Pubmed database search: 621 results

| # | Concept | Searches | Results |
| --- | --- | --- | --- |
| 1 | Overweight/obesity | "Obesity"[Mesh] OR "Overweight"[Mesh] OR "obesity"[tw] OR "obese"[tw] OR "obese patien*"[tw] OR "overweight"[tw] OR "overweight*"[tw] OR "overweightness"[tw] OR "Body Mass Index"[Mesh] OR "body weight"[Mesh] OR "Body Mass Index"[tw] OR "BMI"[tw] OR "body weight"[tw] | 989,688 |
| 2 | Type 2 diabetes mellitus | "Diabetes Mellitus, Type 2"[Mesh] OR "type 2 diabetes mellitus"[tw] OR "type 2 diabetes"[tw] OR "t2dm"[tw] OR "non-insulin-dependent diabetes mellitus"[tw] OR "NIDD"[tw] | 216,133 |
| 3 | Prevalence/associated factors | "Prevalence"[Mesh] OR "Prevalence"[tw] OR "prevalen*"[tw] OR "risk factors"[Mesh] OR "risk factors"[tw] OR "association"[Mesh] OR "associati*"[tw] OR "associate"[tw] OR "precipitating factors" [Mesh] OR "precipitating factors" [tw] OR "precipitat*"[tw] | 3,429,363 |
| 4 | Africa | "Africa"[Mesh] OR "Africa"[tw] OR "Africa, central"[Mesh] OR "Africa, eastern"[Mesh] OR "Africa, northern"[Mesh] OR "Africa, southern"[Mesh] OR "Africa, western"[Mesh] | 360,447 |
| 5 | #1 AND #2 AND #3 AND #4 | ((("Obesity"[Mesh] OR "Overweight"[Mesh] OR "obesity"[tw] OR "obese"[tw] OR “obese patien*"[tw] OR "overweight"[tw] OR "overweight*"[tw] OR "overweightness"[tw] OR "Body Mass Index"[Mesh] OR "body weight"[Mesh] OR "Body Mass Index"[tw] OR "BMI"[tw] OR "body weight"[tw]) AND ("Diabetes Mellitus, Type 2"[Mesh] OR "type 2 diabetes mellitus"[tw] OR "type 2 diabetes"[tw] OR "t2dm"[tw] OR "non-insulin-dependent diabetes mellitus"[tw] OR "NIDD"[tw])) AND ("Prevalence"[Mesh] OR "Prevalence"[tw] OR "prevalen*"[tw] OR "risk factors"[Mesh] OR "risk factors"[tw] OR "association"[Mesh] OR "associati*"[tw] OR "associate"[tw] OR "precipitating factors" [Mesh] OR "precipitating factors" [tw] OR "precipitat*"[tw])) AND ("Africa"[Mesh] OR "Africa"[tw] OR "Africa, central"[Mesh] OR "Africa, eastern"[Mesh] OR "Africa, northern"[Mesh] OR "Africa, southern"[Mesh] OR "Africa, western"[Mesh]) | 659 |
| 6 | #1 AND #2 AND #3 AND #4 Filters: from 2000 - 2022 |  | 621 |

Embase database search: 700 results

| # | Concept | Searches | Results |
| --- | --- | --- | --- |
| 1 | Overweight/obesity | exp obesity/ OR overweight.mp. OR exp body mass/ OR BMI$.mp. OR body mass index.mp. OR Obese patien.mp. | 1092680 |
| 2 | Type 2 diabetes mellitus | exp non insulin dependent diabetes mellitus/ OR type 2 diabetes mellitus.mp. OR type 2 diabetes.mp. OR T2DM$.mp. OR NIDD$.mp. | 353723 |
| 3 | Prevalence/associated factors | exp prevalence/ OR exp risk factor/ OR predictors.mp. OR exp disease predisposition/ | 2563767 |
| 4 | Africa | exp Africa/ | 379715 |
| 5 |  | 1 AND 2 AND 3 AND 4 | 717 |
| 6 |  | Limit 5 to yr=‘2000- 2022’ | 700 |

MEDLINE database search: 263 results

| # | Concept |  |  |
| --- | --- | --- | --- |
| 1 | Overweight/obesity | exp Obesity/ OR exp Overweight/ OR exp body mass index/ OR BMI$.mp. OR Obese patien$.mp. | 435169 |
| 2 | Type 2 diabetes mellitus | exp Diabetes Mellitus, Type 2/ OR type 2 diabetes mellitus.mp. OR non insulin dependent diabetes mellitus.mp. OR NIDD$.mp. OR T2DM$.mp. | 183559 |
| 3 | Prevalence/associated factors | exp prevalence/ OR exp risk factors/ OR predictor.mp. OR exp causality/ OR exp precipitating factors/ OR predisposing factors.mp. | 1381228 |
| 4 | Africa | exp Africa/ | 307274 |
| 5 |  | 1 AND 2 AND 3 AND 4 | 281 |
| 6 |  | Limit 5 to yr=‘2000- 2022’ | 263 |

Africa Index Medicus (AIM) database search: 155 results

tw:((tw:(obesity)) OR (tw:(overweight)) OR (tw:(body mass index)) OR (tw:(bmi)) AND (tw:(type 2 diabetes mellitus)) OR (tw:(non-insulin-dependent diabetes mellitus)) OR (tw:(t2dm)))

**Appendix 2.**

JBI Checklist for Analytical Cross-Sectional Studies

| First Author (Year) | Were the criteria for inclusion in the sample clearly defined? | Were the study subjects and the setting described in detail? | Was the exposure measured in a valid and reliable way? | Were objective, standard criteria used for measurement of the condition? | Were confounding factors identified? | Were strategies to deal with confounding factors stated? | Were the outcomes measured in a valid and reliable way? | Was appropriate statistical analysis used? | Total results |
| --- | --- | --- | --- | --- | --- | --- | --- | --- | --- |
| Sinamaw et al (2022) | Yes | Yes | Yes | Yes | No | No | Yes | Yes | 6 |
| Bizuayehu et al., (2022) | Yes | Yes | Yes | Yes | No | Yes | Yes | Yes | 7 |
| Bideberi and Mutagaywa,(2022) | Yes | Yes | Yes | Yes | No | Yes | Yes | Yes | 7 |
| Ebrahim et al., (2022) | Yes | Yes | Yes | Yes | No | No | Yes | Yes | 6 |
| Chetoui et al., (2022) | Yes | Yes | Yes | Yes | No | No | Yes | Yes | 6 |
| Seid et al., (2022) | Yes | Yes | No | Yes | No | No | Yes | Yes | 5 |
| Abebe et al., (2022) | Yes | Yes | Yes | Yes | No | No | Yes | Yes | 6 |
| Abera et al., (2022) | Yes | Yes | No | Yes | No | Yes | Yes | Yes | 6 |
| Umelo et al., (2022) | Yes | Yes | Yes | Yes | No | No | Yes | Yes | 6 |
| Junaid et al., (2022) | Yes | Yes | Yes | Yes | No | No | Yes | Yes | 6 |
| Yusuf et al., (2022) | Yes | Yes | Yes | Yes | No | No | Yes | Unclear | 5 |
| Sebai et al., (2022) | Yes | Yes | Yes | Yes | No | No | Yes | Yes | 6 |
| Kebede et al., (2021) | Yes | No | Yes | Yes | No | No | Yes | Yes | 5 |
| Ibrahim et al., (2021) | Yes | Yes | Yes | Yes | No | No | Yes | Unclear | 5 |
| Yosef et al., (2021) | Yes | Yes | Yes | Yes | No | Yes | Yes | Yes | 7 |
| Omar et al., 2021 | Yes | Yes | Yes | Yes | No | No | Yes | Yes | 6 |
| Djonor et al., (2021) | Yes | Yes | Yes | Yes | No | No | Yes | Yes | 6 |
| Saasita et al., (2021) | Yes | Yes | Yes | Yes | No | No | Yes | Yes | 6 |
| Abdissa et al., (2021) | Yes | Yes | Yes | Yes | No | No | Yes | Yes | 6 |
| Tino et al., (2020) | No | Yes | Yes | Yes | Yes | Yes | Yes | Yes | 7 |
| Otieno et al., (2020) | Yes | Yes | Yes | Yes | No | No | Yes | Yes | 6 |
| Zerga and Bezabih, (2020) | Yes | Yes | Yes | Yes | Yes | Yes | Yes | Yes | 8 |
| Munyogwa et al., (2020) | Yes | Yes | Yes | Yes | No | No | Yes | Yes | 6 |
| Taderegew (2020) | Yes | No | Yes | Yes | No | Yes | Yes | Yes | 6 |
| Haile and Timerga, (2020) | Yes | Yes | Yes | Yes | Yes | Yes | Yes | Yes | 8 |
| Akalu and Birhen (2020) | Yes | Yes | Yes | Yes | No | Yes | Yes | Yes | 7 |
| Kouitcheu et al., (2020) | Yes | Yes | Yes | Yes | No | No | Yes | Yes | 6 |
| Achila et al., (2020) | Yes | Yes | Yes | Yes | No | No | Yes | Yes | 6 |
| Abdissa et al., (2020) | Yes | Yes | Yes | Yes | No | Yes | Yes | Yes | 7 |
| Mohamed et al., (2019) | Yes | No | Yes | Yes | No | No | Yes | Yes | 5 |
| Bello-Ovosi et al., (2019) | Yes | Yes | Yes | Yes | No | No | Yes | Yes | 6 |
| Karau et al., (2019) | Yes | Yes | Yes | Yes | No | No | Yes | Yes | 6 |
| Anioke et al., (2019) | Yes | Yes | Yes | Yes | No | No | Yes | Yes | 6 |
| Kiros et al., (2019) | Yes | No | Yes | Yes | No | No | Yes | Yes | 5 |
| Asamoah-Boaheng et al., (2019) | Yes | Yes | Yes | Yes | No | Yes | Yes | Yes | 7 |
| Gezawa et al., (2019) | Yes | No | Yes | Yes | No | No | Yes | Yes | 5 |
| Bekele et al., (2019) | Yes | Yes | Yes | Yes | No | Yes | Yes | Yes | 7 |
| Fekadu et al., (2019) | Yes | Yes | No | Yes | No | No | Yes | Yes | 5 |
| Kasimu and Rahman, (2019) | Yes | No | Yes | Yes | No | No | Unclear | Yes | 4 |
| Shiriyedeve et al., (2019) | Yes | Yes | Yes | Yes | No | No | Yes | Yes | 6 |
| Bouhajja et al., (2018) | Yes | No | Yes | Yes | Yes | Yes | Yes | Yes | 7 |
| Sarfo-Kantanka et al., (2018) | Yes | Yes | Yes | Yes | No | No | Yes | Yes | 6 |
| Wolde et al., (2018) | Yes | Yes | Yes | Yes | No | No | Yes | Yes | 6 |
| Balogun et al., (2018) | Yes | Yes | Yes | Yes | No | No | Yes | Yes | 6 |
| Birkinshaw et al., (2018) | Yes | No | Yes | Yes | No | No | Yes | Yes | 5 |
| Damian et al., (2017) | Yes | Yes | Yes | Yes | No | No | Yes | Yes | 6 |
| Otieno et al., (2017) | Yes | Yes | Yes | Yes | No | No | Yes | Yes | 6 |
| Ojieabu et al., (2017) | Yes | Yes | No | Yes | No | No | No | Yes | 4 |
| Ali et al., (2017) | Yes | Yes | Yes | Yes | No | No | Yes | Yes | 6 |
| Mwanri et al., (2017) | Yes | Yes | No | Yes | No | No | No | Yes | 4 |
| Goie and Naidoo (2016) | Yes | Yes | Yes | Yes | No | No | Yes | Yes | 6 |
| Habtewold et al., (2016) | Yes | Yes | Yes | Yes | Yes | No | Yes | Yes | 7 |
| Mogre et al., (2016) | Yes | Yes | Yes | Yes | No | No | Yes | Yes | 6 |
| Belkacemi et al., (2016) | No | No | Yes | Yes | No | No | Yes | Yes | 4 |
| Adebola et al., (2016) | Yes | Yes | Yes | Yes | No | No | Yes | Yes | 6 |
| Diaf et al., (2015) | Yes | Yes | Yes | Yes | No | No | Yes | Yes | 6 |
| Adeniyi et al., (2015) | Yes | Yes | Yes | Yes | No | Yes | Yes | Yes | 7 |
| Camara et al., (2015) | Yes | Yes | Yes | Yes | No | No | Yes | Yes | 6 |
| Ndege et al., (2014) | Yes | Yes | No | Yes | No | Yes | Yes | Yes | 6 |
| Kamuhabwa and Charles (2014) | Yes | Yes | Yes | Yes | No | Yes | Yes | Yes | 7 |
| Mogre et al., (2014) | Yes | Yes | Yes | Yes | No | No | Yes | Yes | 6 |
| Brenyah et al., (2013) | Yes | Yes | Yes | Yes | No | No | Yes | Yes | 6 |
| Okafor and Ofoegbu (2012) | No | No | Yes | Yes | No | No | Yes | Yes | 4 |
| Berraho et al., (2012) | No | No | Yes | Yes | No | No | Yes | Yes | 4 |
| Acquah et al., (2011) | Yes | Yes | No | Yes | No | No | Yes | Yes | 5 |
| Baba et al., (2010) | Yes | Yes | Unclear | Yes | No | No | Yes | Yes | 5 |
| Elnasri and Ahmed (2008) | Yes | Yes | Yes | Yes | No | No | Yes | Yes | 6 |
| Alshkri and Elmehdawi (2008) | No | No | Yes | Yes | No | No | Yes | Yes | 4 |
| Ajayi et al., (2009) | Yes | No | No | Yes | No | No | Yes | Yes | 4 |
| Choukem et al., (2007) | Yes | Yes | Yes | Yes | No | No | Yes | Yes | 6 |
| Akande et al., (2007) | Yes | No | Yes | Yes | No | No | Yes | Unclear | 4 |
| Fasanmade and Okubadejo, (2007) | No | No | Yes | Yes | No | No | Yes | Yes | 4 |
| Adediran et al., (2007) | Yes | Yes | Yes | Yes | No | No | Yes | Unclear | 5 |
| Makuyana et al., (2004) | Yes | No | Yes | Yes | No | No | Yes | Yes | 5 |

JBI Checklist for Case Control Studies

| First Author (Year) | Were the groups comparable other than the presence of disease in cases or the absence of disease in controls? | Were cases and controls matched appropriately? | Were the same criteria used for identification cases and controls? | Was exposure measured in a standard, valid and reliable way? | Was exposure measured in the same way for cases and control? | Were confounding factors identified? | Were strategies to deal with confounding factors stated? | Were outcomes assessed in a standard, valid and reliable way for cases and control? | Was the exposure period of interest long enough to be meaningful? | Was appropriate statistical analysis used? | Total score |
| --- | --- | --- | --- | --- | --- | --- | --- | --- | --- | --- | --- |
| Shigidi etal., (2021) | Yes | Yes | Yes | Yes | Yes | No | No | Yes | Yes | Yes | 8 |
| Kotiso et al., (2021) | Yes | No | Yes | Yes | Yes | No | Yes | Yes | Yes | Yes | 8 |
| Ekoru et al., (2019) | No | Unclear | Yes | Yes | Yes | Yes | Yes | Yes | Yes | Yes | 8 |
| Gudjinu and Sarfo, (2017) | Yes | Unclear | Yes | Yes | Yes | Yes | Yes | Yes | Yes | Yes | 9 |
| Danquah et al., (2012) | No | Unclear | Yes | Yes | Yes | No | Yes | Yes | Yes | Yes | 7 |

JBI Critical Appraisal Checklist for Cohort Studies

| Author | Were the two groups similar and recruited from the same population? | Were the exposures measured similarly to assign people to both exposed and unexposed group? | Was the exposure measured in a valid and reliable way? | Were confounding factors identified? | Were strategies to deal with confounding factors stated? | Were the groups/  participants free of the outcome at the start of the study (or at the moment of exposure)? | Were the outcomes measured in a valid and reliable way? | Was the follow up time reported and sufficient to be long enough for outcomes to occur? | Was follow up complete, and if not, were the reasons to loss to follow up described and explored? | Were strategies to address incomplete follow utilized? | Was appropriate statistical analysis used? | Total score |
| --- | --- | --- | --- | --- | --- | --- | --- | --- | --- | --- | --- | --- |
| Regassa and Tola (2021) | Unclear | Unclear | Yes | No | No | Yes | Yes | Yes | Yes | No | Yes | 6 |

**Appendix 3**

Summary statistics of meta-regression of the prevalence of overweight and obesity among T2DM patients in Africa.

| **Variables** | **Estimate** | **Standard error** | **Z value** | **P value** | **95% CI lower limit** | **95% CI upper limit** |
| --- | --- | --- | --- | --- | --- | --- |
| Year of publication | 0.0089 | 0.0201 | 0.4419 | 0.6585 | 0.0306 | 0.0484 |
| Male to female ratio | -0.2237 | 0.258 | -0.8669 | 0.386 | -0.7294 | 0.2820 |
| Mean age of participants | 0.034 | 0.0236 | 1.444 | 0.1486 | -0.0121 | 0.0802 |
| Study settings: Single site vs multi-site facility | 0.2618 | 0.216 | 1.2117 | 0.2256 | -0.1617 | 0.6852 |
| BMI assessment (No vs Yes) | 0.0525 | 0.2143 | 0.245 | 0.8064 | -0.3675 | 0.4725 |
| Time since diagnosis | 0.0343 | 0.0819 | 0.4188 | 0.6754 | -0.1263 | 0.1949 |
| Geographical region |  |  |  |  |  |  |
| Central vs East Africa | 0.2804 | 0.4644 | 0.6039 | 0.5459 | -0.6298 | 1.1907 |
| North vs East Africa | 0.5939 | 0.3006 | 1.9756 | 0.0482 | 0.0047 | 1.1830 |
| Southern vs East Africa | 1.7606 | 0.3483 | 5.0554 | <0.0001 | 1.078 | 2.4432 |
| West vs East Africa | 0.1888 | 0.1709 | 1.1043 | 0.2694 | -0.1463 | 0.5238 |
| Sampling strategy: probability vs non-probability | -0.7187 | 0.2237 | -3.2125 | 0.0013 | -1.1572 | -0.2802 |
| Total sample size | -0.0001 | 0.00 | -1.3457 | 0.1784 | -0.0002 | 0.00 |

**Appendix 4**


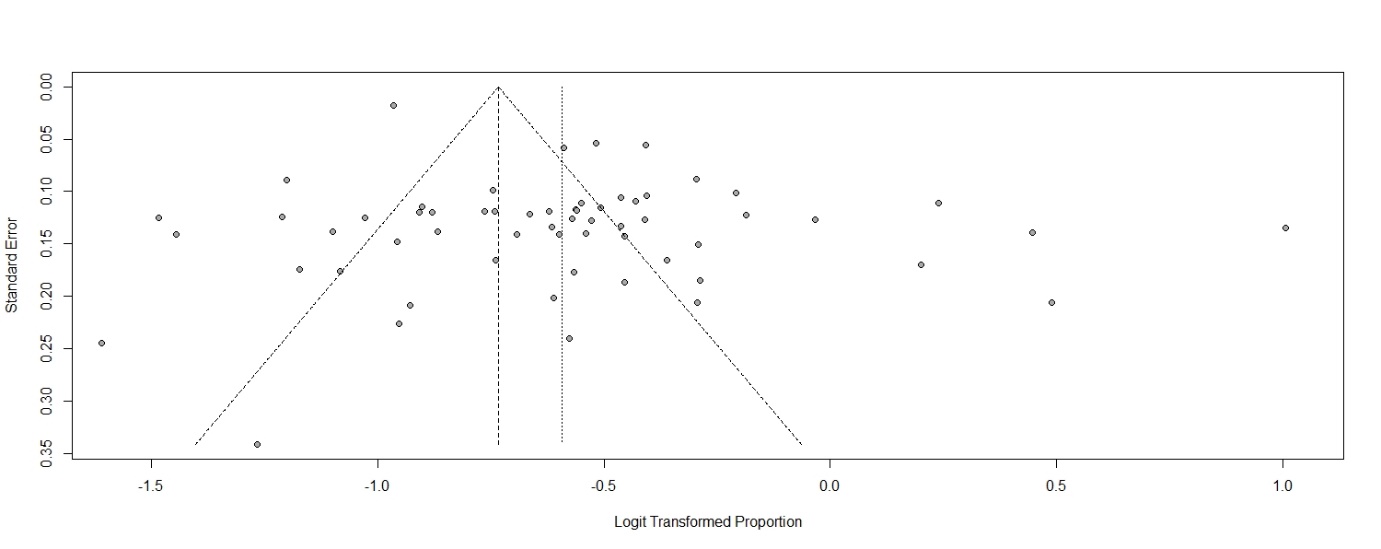


Funnel plot for studies on the prevalence of overweight among T2DM patients in Africa.


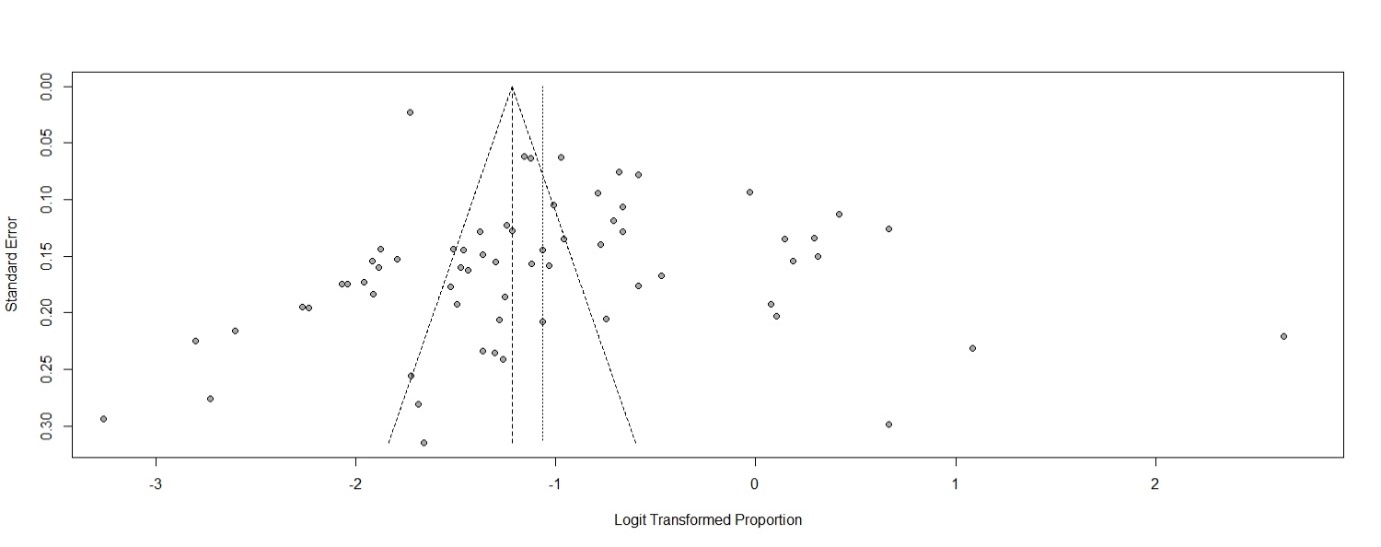
Funnel plot for studies on the prevalence of obesity among T2DM patients in Africa.


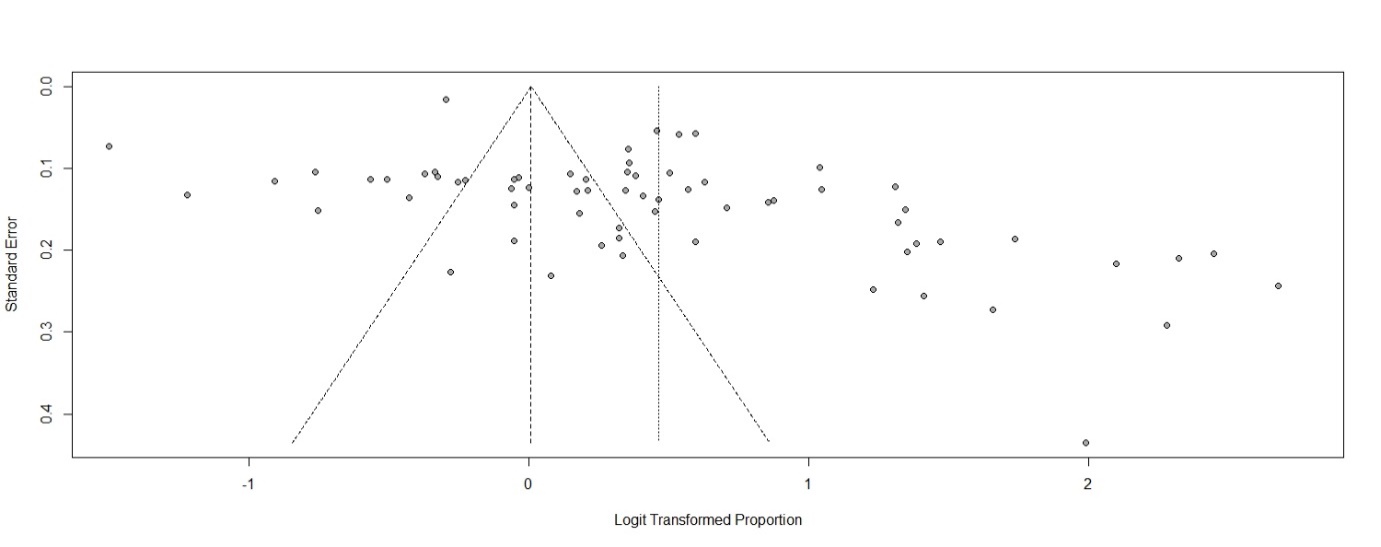


Funnel plot for studies on the prevalence of both overweight and obesity among T2DM patients in Africa.

Egger’s test result for studies on overweight prevalence: t = 3.48, df = 58, p-value = 0.0010

Egger’s test result for studies on obesity prevalence: t = 2.59, df = 63, p-value = 0.0120

Egger’s test results for studies on both overweight and obesity prevalence: t = 5.02, df = 65, p-value < 0.0001
